# Supplementary material for: Game-theoretic agent-based modelling of micro-level conflict: Evidence from the ISIS-Kurdish war
Source: PLoS One. 2024 Jun 5;19(6):e0297483. doi: 10.1371/journal.pone.0297483 (PMC11152260; doi:10.1371/journal.pone.0297483)
Supplement: S1 Table — (PDF) [file pone.0297483.s001.pdf]

S1 Table: Game Theory strategies from the literature.

Table 1. Game Theory Strategies from the Literature

Source	Strategy	Name	Description	Included?
[1]	Tit for Tat	TFT	Begin by cooperating, then copy opponent's last move	✓
[1]	Tideman and Chieruzzi	T&C	Begins with TFT. An extra punishment is then added after each of the opponent's round of defections. There are a number of conditions under which the opponent is given a fresh start	✗
[1]	Nydegger	NY	Begins with TFT (with some exceptions for third move), after which its choice is determined by $A = 16a_1 + 4a_2 + a_3$	✗
[1]	Grofman	GRO	If each played a different move in the previous turn, cooperates with probability 2/7, otherwise always cooperates	✓
[1]	Shubik	SH	Cooperates until opponent defects, after which the length of retaliation increases by one for each departure from mutual cooperation	✗
[1]	Stein and Rapoport	S&R	Cooperates on first four moves, then plays TFT. Every fifteen moves checks if opponent is playing randomly, and defects last two moves	✗
[1]	Grudger	GRU	Starts by cooperating but will forever defect after the first defection by the opponent	✗
[1]	Davis	DA	Cooperates for ten rounds, then plays Grudger	✗
[1]	Graaskamp	GRA	TFT for first fifty moves, defects on move 51, then five more moves of TFT. If opponent seems random, defect forever, if TFT or Analogy, plays TFT, otherwise defects every 5-15 moves	✗
[1]	First by Downing	DO	Assumes opponent cooperates with fixed probability, continuously updated. Plays to maximise its own payoff	✗
[1]	Feld	FE	Starts with TFT, then gradually lowers its probability of cooperation to 0.5 by 200th move	✗
[1]	Joss	JO	Cooperates 90% of the time after opponent's cooperation, always defects after opponent's defection	✗
[1]	Tullock	TU	Cooperates on first eleven moves, then cooperates 10% less than opponents has cooperated on preceding ten moves	✗
[1]	(Name withheld)	NA	Cooperation probability is uniformly random in the 30-70% range	✗
[1]	Tit for 2 Tats	TF2T	Starts by cooperating, then defects only after two defections by opponent	✓
[1]	Go by majority	GM	Examines the history of the opponent: if the opponent has more defections than cooperations then the player defects. In case of equal number of defections and cooperations this player will cooperate	✗
[2]	Always cooperate	COP	Always cooperates	✓
[2]	Always defect	DEF	Always defects	✓
[2]	Extort-2	EX	Extortionate Zero Determinant Strategy with l=P	✗
[2]	Hard tit for tat	HTFT	Variant of TFT with longer history for retaliation	✓
[2]	Generous tit for tat	GTFT	Variant of TFT with longer history for retaliation	✓
[2]	Win-stay-lose-shift	WSLS	Cooperates in response to cooperation, but retaliates with sustained defection	✗
[3]	Calculator	CAL	Plays like Joss for first 20 rounds. If periodic behavior is detected, defect forever, otherwise play TFT	✗
[3]	Prober	PR	Plays DCC initially. Defects forever if opponent cooperated in moves 2 and 3., otherwise plays TFT	✗
[3]	Prober 2	PR2	Plays DCC initially. Cooperates forever if opponent played D then C in moves 2 and 3. Otherwise plays TFT	✗
[3]	Hard prober	HPR	Plays DDCC initially. Defects forever if opponent cooperated in moves 2 and 3. Otherwise plays TFT	✗

References

1. Axelrod R. More effective choice in the prisoner's dilemma. The Journal of Conflict Resolution. 1980;24(3):379–403.

2. Stewart AJ, Plotkin JB. Extortion and cooperation in the Prisoner's Dilemma. Proceedings of the National Academy of Sciences. 2012;109(26):10134–10135.

3. Press W, Dyson F. Iterated Prisoner's Dilemma contains strategies that dominate any evolutionary opponent. Proceedings of the National Academy of Sciences. 2012;109(26):10409–10413.
