# Supplementary material for: Game-theoretic agent-based modelling of micro-level conflict: Evidence from the ISIS-Kurdish war
Source: PLoS One. 2024 Jun 5;19(6):e0297483. doi: 10.1371/journal.pone.0297483 (PMC11152260; doi:10.1371/journal.pone.0297483)
Supplement: S2 Table — (PDF) [file pone.0297483.s002.pdf]

S2 Table: Additional Game Theory strategies.

Table 2. Additional Game Theory Strategies [1]

Strategy	Name	Description	Included?
Aggravator	AGG	Grudger, except that it defects on the first 3 turns	✗
Alternator	ALT	Alternates between cooperating and defecting	✓
2 tits for tat	2TFT	Starts by cooperating and retaliates to each defection with two defections	✓
Backstabber	BS	Forgives the first 3 defections but on the fourth will defect forever. Defects on the last 2 rounds unconditionally	✗
Bully	BU	Opposite to TFT, including first move	✓
Cycler CCD	CCD	Repeats sequence CCD indefinitely	✓
Cycler CCCD	CCCD	Repeats sequence CCCD indefinitely	✓
Cycler DDC	DDC	Repeats sequence DDC indefinitely	✓
Adaptive	AD	Start with a specific sequence of C and D, then play the strategy that has worked best, recalculated each turn	✗
Desperate	DES	Only cooperates after mutual defection	✓
Doubler	DOU	Cooperates except when the opponent has defected and the opponent's cooperation count is less than twice their defection count	✗
Easy go	EG	Starts by defecting however will cooperate if at any point the opponent has defected	✗
Firm but fair	FBF	Cooperates on the first move, and cooperates except after receiving a sucker payoff	✓
Forgiver	FOR	Starts by cooperating, but will defect if at any point the opponent has defected more than 10 percent of the time	✗
Fool me once	FMO	Forgives one defection then retaliates forever on a second defection	✗
Forgetful grudger	FG	Starts by cooperating however will defect if at any point the opponent has defected, but forgets after a given number of turns	✓
Grudger alternator	GA	Starts by cooperating until the first opponent's defection, then alternates DC	✗
Handshake	HS	Starts with CD. If the opponent plays the same way, cooperate forever, else defect forever	✗
Hopeless	HL	Only defects after mutual cooperation	✓
Negation	NG	Starts by cooperating or defecting randomly if it's their first move, then simply doing the opposite of the opponents last move thereafter	✗
Nice average copier	NAC	Starts by cooperating, then cooperates with probability p if the opponent's cooperation ratio is p	✗
Opposite grudger	OP	Starts by defecting but will cooperate if at any point the opponent has cooperated	✗
Soft grudger	SG	Instead of punishing by always defecting, punishes by playing: DDDDCC (continues to cooperate afterwards)	✓
Tricky co-operator	TC	Cooperator that is trying to be tricky (defects once in a while)	✗
Tricky defector	TD	Defector that is trying to be tricky (cooperates once in a while)	✗
Willing	WL	Only defects after mutual defection	✓
Appeaser	APP	Tries to guess what the opponent wants. Switch the classifier every time the opponent plays D. Start with C, switch between C and D when opponent plays D	✗
A Pavlov 2006	PAV	Attempts to classify opponent into Cooperative, ALLD, STFT, PavlovD, or Random and tries to achieve mutual cooperation or defects against uncooperative moves	✗
Usually cooperates	UC	Cooperates except after a C following a D	✓
Usually defects	UD	Defects except after a D following a C	✓

References

1. Knight V, Campbell O, Harper M, Langner K, Campbell J, Campbell T, et al. An Open Framework for the Reproducible Study of the Iterated Prisoner's Dilemma. Journal of Open Research Software. 2016;4:1–11.
