# Supplementary material for: Game-theoretic agent-based modelling of micro-level conflict: Evidence from the ISIS-Kurdish war
Source: PLoS One. 2024 Jun 5;19(6):e0297483. doi: 10.1371/journal.pone.0297483 (PMC11152260; doi:10.1371/journal.pone.0297483)
Supplement: S3 Table — Resulting patterns of defection after playing strategies against all others and itself in an Iterated Prisoner’s Dilemma game using the Axelrod library. (PDF) [file pone.0297483.s003.pdf]

S3 Table: Strategy categorisation.

Resulting patterns of defection after playing strategies against all others and itself in an Iterated Prisoner's Dilemma game using the Axelrod library.

Table 3. Strategy Categorisation.

	TFT	TF2T	COP	DEF	HTFT	ALT	2TFT	BU	CCD
TFT	-	-	-	abab	-	abab	-	abab	abab
TF2T	-	-	-	abab	-	bbb	-	ababb	bbb
COP	-	-	-	bbb	-	bbb	-	bbb	bbb
DEF	abab	abab	aaa	abab	abab	aab	abab	aaa	aaab
HTFT	-	-	-	abab	-	aab	-	babaaa	aaab
ALT	abab	aaa	aaa	abb	abb	abab	abb	abab	aabab
2TFT	-	-	-	abab	-	aab	-	aabab	aab
BU	aabb	aaabb	aaa	bbb	aabbbb	abab	aabbb	abab	aab
CCD	abab	aaa	aaa	abbb	abbb	babab	abb	abb	abab
CCCD	abab	aaa	aaa	abbbb	abbb	abb	abb	abbb	abababb
DDC	aabb	aab	aaa	ababb	ababb	aabababb	ababb	aab	aab
DES	aabb	aaabb	aaa	abb	abb	abab	abb	abab	aab
FBF	-	-	-	abab/bbb	-	abab	-	baba/babb	abab
HL	abab	aaa	aaa	bbb	aaab	abab	abb	abab	abab
WL	-	-	-	abab	-	bbb	-	bbb	bbb
UC	-	-	-	bbb	-	abab	-	bbb	abab
UD	aabb	abab	aaa	abab	abab	abab	abab	aaa	aaab

	CCCD	DDC	DES	FBF	HL	WL	UC	UD
TFT	abab	abab	abab	-	abab	-	-	abab
TF2T	bbb	abb	ababb	-	bbb	-	-	abab
COP	bbb	bbb	bbb	-	bbb	-	-	bbb
DEF	aaaab	aabab	aab	aab	aaa	abab	aaa	abab
HTFT	aaab	aabab	aab	-	aaab	-	-	abab
ALT	aab	abababb	abab	abab	abab	aaa	abab	abab
2TFT	aab	aabab	aab	-	aab	-	-	abab
BU	aaab	abb	abab	aabb	aaa	aaa	aaa	bbb
CCD	abababa	abb	abb	abab	abab	aaa	abab	abbb
CCCD	abab	2(abbb)abb	abbb	abab	abb	aaa	abab	abbb
DDC	2(aaab)aab	abab	abab	aab	aab	aaa	aaa	abab
DES	aaab	aabb	abab	aab/aabb	aaa	aaa	aaa	abab
FBF	abab	baba/babb	baba/babb	-	abab	-	-	baba/bab
HL	aab	abb	abab	abab	abab	aaa	abab	bbb
WL	bbb	bbb	bbb	-	bbb	-	-	abab/bbb
UC	abab	bbb	bbb	-	abab	-	-	bbb
UD	aaab	aabb	abab	/	aaa	abab/aaa	aaa	abab
