# Supplementary material for: Game-theoretic agent-based modelling of micro-level conflict: Evidence from the ISIS-Kurdish war
Source: PLoS One. 2024 Jun 5;19(6):e0297483. doi: 10.1371/journal.pone.0297483 (PMC11152260; doi:10.1371/journal.pone.0297483)
Supplement: S1 Appendix — (PDF) [file pone.0297483.s004.pdf]

S1 Appendix: Data extraction and pre-processing.

Data was retrieved from the ACLED website via `https://acleddata.com/data-export-tool/`. Access is free; however it requires registration – for that reason the dataset itself has not been included here. To replicate the dataset used in this analysis, the fields used for the data retrieval were:

From: 01/01/2017
To: 31/12/2019
Event Type: Battles, Explosions/Remote Violence
Sub Event Type: All
Actor Type: All
Actor: All
Region: Middle East
Country: Syria
Location: All
Keyword: N.A.
Export Type: Compatibility Mode

Once the dataset had been retrieved, only events including relevant actors were kept. This included actors in the both the 'actor1' and 'actor2' columns. Below is a list of all actors any event containing one of these was kept, while the rest were removed.

Kurdish forces:

- Global Coalition Against Daesh
- QSD: Syrian Democratic Forces
- YPG: Peoples Protection Units - Special Task Forces
- YPG: Peoples Democratic Forces - Anti-Terror Unit
- YPG: Peoples Protection Units
- YPG: Peoples Protection Units - Anti-Terror Unit
- YPG: Peoples Protection Units - Hezen Komandoz
- YPJ: Women's Protection Units
- YPS: Civil Protection Units
- YRK: Eastern Kurdistan Units
- TAK: Kurdistan Freedom Hawks
- PKK-YJA STAR: Kurdistan Workers Party-YJA STAR
- PKK: Kurdistan Workers Party

Islamic State forces:

- Islamic State (Syria)
- Islamic State (Iraq)

To determine the locations that would be modelled, the data was then grouped using the 'location' column. These locations were then ordered by total number of events recorded, and those with 50 or more locations (we confirmed all were within the Rojava region) were ultimately used for the analysis (see Materials and Methods section).

For later steps of the analysis, the data was split into each location and quarter, creating a new CSV file for each segment.

Code for replication of the analysis is available on this GitHub Repository: `https://github.com/oliviams/Rojava_2022`.
