# Supplementary material for: Game-theoretic agent-based modelling of micro-level conflict: Evidence from the ISIS-Kurdish war
Source: PLoS One. 2024 Jun 5;19(6):e0297483. doi: 10.1371/journal.pone.0297483 (PMC11152260; doi:10.1371/journal.pone.0297483)
Supplement: S2 Appendix — (PDF) [file pone.0297483.s005.pdf]

# S2 Appendix: Implementation of Axelrod library.

Each of the selected strategies in the iterated Prisoner's Dilemma game were played against all others and itself using the Axelrod Python Library [1]. For example, to play ALT against TFT for 10 rounds, we would use the following:

```
import axelrod
strategies = [axelrod.TitForTat(), axelrod.Alternator()]
match = axelrod.Match(strategies, 10)
interactions = match.play()
print(interactions)
```

This would give the following output (C denotes cooperation and D is defection):

```
[(C, C), (C, D), (D, C), (C, D), (D, C), (C, D), (D, C), (C, D),
(D, C), (C, D)]
```

The pattern of defection extracted from this is *bababababa*, where the recurring pattern would then be *abab*.

## References


1. Knight V, Campbell O, Harper M, Langner K, Campbell J, Campbell T, et al. An Open Framework for the Reproducible Study of the Iterated Prisoner's Dilemma. Journal of Open Research Software. 2016;4:1–11.
