# Supplementary material for: Game-theoretic agent-based modelling of micro-level conflict: Evidence from the ISIS-Kurdish war
Source: PLoS One. 2024 Jun 5;19(6):e0297483. doi: 10.1371/journal.pone.0297483 (PMC11152260; doi:10.1371/journal.pone.0297483)
Supplement: Attachment — Submitted filename: Rebuttal Letter.docx [file pone.0297483.s006.docx]

**Rebuttal Letter**

The authors would like to thank the reviewers for their fair reviews and detailed list of constructive suggestions. We greatly appreciate the opportunity to resubmit our revised paper for your renewed consideration. Below we respond to both reviewers’ comments and point to the corresponding changes we made in the paper. We mention the lines where these changes have been made, and have also submitted the new version of the manuscript with these changes highlighted.

## Reviewer 1

*1. The research question should be written explicitly.*

Thank you for this comment, as indeed the question was not clear enough from the outset. We have clarified what exactly we set out to study in lines 11-14.

*2. The text on pages 1 and 2 is not easy to follow. They need to be edited.*

We have made some changes, in particular relating to the points raised by the reviewers which we address in the relevant points (1-6). If there are any additional sections that remain unclear, we will endeavour to further clarify these.

*3. The first sentence of the second paragraph, "The systematic study of civil conflict is a* *remarkably recent field," could be criticized for being too generalizing. While it is true that certain approaches to the study of civil conflict are relatively new, scholars have been studying civil conflict for decades, if not centuries, in various forms. It might be more accurate to say that certain subfields or methods of studying civil conflict are recent developments.*

While it is true that there has been some study on civil war dates back decades, the increased number of civil conflicts in recent years has sparked widespread interest – some have attributed the spark in interest to the conflicts cause by the fall of the Soviet Union (see Huntington, 1996 (The Clash of Civilizations and the Remaking of World Order); Kaplan, 1994 (The Coming Anarchy); Posen, 1993 (The Security Dilemma and Ethnic Conflict)). We have also clarified in the manuscript (line 15) that the notion was taken from Kalyvas (2007 – Civil Wars) – while we had previously cited this chapter, we have now explicitly mentioned that this remark is supported by current conflict scholars.

*4. Lines 5-6: “with regard to conflict dynamics, research has almost exclusively relied on artificial societies”- This argument needs support.*

We previously failed to specify here that this applies mainly to agent-based models, as opposed to statistical models for instance; this has now been made explicit in the manuscript. Having said that, we are referring to conflict dynamics, rather than conflict onset for instance, and while there has been more extensive modelling on the latter (particularly statistical), the dynamics of conflict have not been studied as in-depth. Up to now, models of conflict dynamics have predominantly been agent-based ones, which, as mentioned, generally rely on (possibly large-scale) artificial societies to study emergent phenomena.

*5. The “Introduction” provides an overview of the various causes of civil conflict that have been proposed by scholars. However, it does not delve into the debates and critiques that exist within each of these approaches. For example, while it is mentioned that some economists argue that the abundance of natural resources is a key driver of civil conflict, there is no discussion of the critiques of this "resource curse" theory, which suggest that the relationship between resources and conflict is more complex than a simple causal link.*

The reason for not delving into the literature on causes of civil conflict as deeply is because the focus of our research is on the dynamics of ongoing conflict, rather than on its onset. We think it is important to give a brief overview of the literature, which is why it is included, but causes of conflict are not directly relevant to our research. However, we do understand that there was some oversimplification in the section highlighted by the reviewer, and have addressed this in lines 19-25.

*6. The “Introduction” could also benefit from more detail on the specific models and approaches that have been used to study civil conflict. While some models are mentioned briefly, such as Epstein's agent-based model, there is little explanation of how these models work or what their limitations are.*

The reason we do not provide more detail on some of the models mentioned is because most of these focus on the causes of conflict, whereas we are interested in conflict dynamics. Additionally, these models generally employ statistical methods, whereas we are using a game-theoretic model, therefore the methods used are not directly relevant to our paper. We have tried to make this clear when discussed existing models.

Epstein’s agent-based model is discussed in more detail in lines 97-104 precisely as it is more relevant to our approach. We briefly mention the method used and some of the findings covered in Epstein’s paper. The main difference is that Epstein’s model uses ‘artificial societies’ – meaning it is theoretical rather than based on empirical data. In contrast, our agent-based model is built on empirical data.

*7. The authors only provided the link to the web site where they downloaded the data. The cleaned and filtered data in CSV file, mentioned in “S1 Appendix” was not provided. Similarly, the code for replication of the analysis is not accessed through the “Github Repository” link in “S1 Appendix”. Both the filtered/cleaned data and the code should be provided to the reviewers for replicating the analysis.*

As mentioned in the supporting information, we are not able to share a CSV file of the pro-processed data due to ACLED’s terms of use – this is precisely why we do not refer to a “cleaned and filtered data in CSV file,” as we cannot have this available for download directly. However, as mentioned, the data is freely available via the link provided and we outline the steps we followed in the pre-processing stage in order to replicate our dataset.

The link to the GitHub repository was provided in the initial submission, we have now added both hyperlink and full URL in case the former does not work. The code is available at: <https://github.com/oliviams/Rojava_2022> (the dates for the last commits can be used as verification that the code was already available for the previous submission).

## Reviewer 2

*1. It is unclear what question the manuscript sets out to answer. At first, I thought that this might be a presentation issue. However, the research question, design, and findings are never fully specified in a way that would allow the reader to understand the value and significance of the research. This recurs throughout the manuscript. In the abstract and introduction, the authors explain that they use the agent-based model, in combination with the data, "to identify patterns of interaction" (p.1) between armed groups. But the manuscript never explains what specifically the authors mean by this. Are they setting out to explain when and where battles are likely to occur? When and where cooperation is likely to occur? Something else?*

*The summary of the findings confirms the problem. The authors explain that "shifts in territorial control are found to be more significant than geographical or temporal variables" (p.1). This begs the questions, for what, with respect to what outcome, is territorial control "found to be more significant"? Or, "the distribution of attacks [is found to be] more relevant than specific patterns of behaviour" (p.1). Again, more relevant for what?*

*Without clarification of the above, it is impossible to situate the manuscript within existing research. For example, when comparing its findings to existing research on civil wars, are the findings counterintuitive? Surprising? Building on but consistent with existing research? Unfortunately I am unable to evaluate any of these questions because the manuscript does not specify things sufficiently to allow a reader to do so.*

*This problem also negatively affects the research itself because the authors are proposing to model some kind of behavior, without ever deliberately explaining or conceptualizing what that behavior consists of in the real world. This leads to the second problem.*

See answer to point 1 above – we have clarified the research question as well as what is meant by ‘behaviour,’ which we understand was not explicit enough in the previous version of the submission.

*2. The theoretical model is not well suited to capturing the empirical problem that the authors want to study. Here, I am a bit hampered by problem #1, above. Assuming that the manuscript intends to do something like study why armed groups engage in battles within a civil war, then the theoretical basis for the modeling choices is tenuous. It is fine to point to concepts of cooperation and defection in the prisoner's dilemma. But what does that mean in the real-world conflict in Syria? If the idea is to predict when and where armed groups will use violence against their enemies, for example, then why is it helpful to model this as a prisoner's dilemma? The literature on conflict - across the social sciences - has a long tradition of modeling the decision to go to war, or to engage in conflict, or to choose to fight vs cooperate as a prisoner's dilemma. But after a war has started, why should we think about individual episodes of violence, of battles or attacks, as being related to the same process?*

As mentioned by the reviewer and in lines 207-217, there is a tradition in the literature to model different aspects on conflict using the (iterated) Prisoner’s Dilemma. Given this long tradition, we believe the suitability of this framework to study conflict could be considered established. Regarding the concern that it is only appropriate for conflict onset and not ongoing conflict, the reviewer does not specify why they believe “the theoretical model is not well suited.” Following the example of past scholarship, we think the use of an iterated Prisoner’s Dilemma framework is well suited to the study of ongoing conflict. Firstly, taking an iterated game with an infinite number of rounds appropriately models a conflict in the sense that actors know the past actions of their opponent, and can thus respond to this, but do not know how long the interaction will last or future actions. In the same way that the Prisoner’s Dilemma has been established as suitable to model conflict onset (as mentioned by the reviewer), we can also model the decision to engage in each individual battle or episode of violence in the same way.

*In addition, the authors seem to be operating under the assumption that battles between Kurdish militias and ISIS can be analyzed in isolation from the rest of the civil war in Syria. It is surprising that the authors do not seem to be aware that they are making this assumption, or at best do not acknowledge it in the manuscript. But it is hard to see how the fighting between these armed groups can be analyzed without reference to other important actors in the war and developments in the fighting. This includes changes over time in the strength and presence of Syrian government forces, Russia forces, US and other coalition partners, Turkish military, other Syrian rebel groups, and so on. It also includes events across all of Syria, which may affect decisions by ISIS or by Kurdish militias in specific local areas in which they operate, and international political events, for example attempts at peace negotiations to end the war, for example the Geneva or Astana processes.*

We do not contend that the Kurdish-ISIS conflict can be taken in isolation from the rest of the conflict – this is precisely why we chose to focus on the region of Rojava in particular. We have made this clearer in lines 142-147 – while we agree with the reviewer that we need to consider the wider conflict and other events that are happening at the same time, we focus on this particular area because of the dyadic nature of this conflict and the fact that the Rojava region has predominantly seen conflict between these two actors.

*3. The manuscript does not engage sufficiently with existing research. First, there are studies of civil war dynamics that use empirical evidence from Syria and which link to questions of conflict and cooperation that the authors discuss. A couple of these are about battlefield dynamics focusing on infighting between rebel groups. They might not speak directly to the battles between Kurdish militias and the Islamic State, but it is important to assess what kinds of theoretical explanations they offer and what light they shed on the war's empirics. Others are directly about the Kurdish-ISIS conflict. See: Schulhofer-Wohl, "On-side fighting in civil war: The logic of mortal alignment in Syria," Rationality and Society (2020); Schwab, "Escalate or negotiate? Constraint and rebel strategic choices towards rivals in the Syrian civil war," Terrorism and Political Violence (2021); van Wilgenburg and Fumerton, "From the PYD-YPG to the SDF: The consolidation of power in Kurdish-controlled northeast Syria," Studies in Conflict and Terrorism (2021); and Yeşiltaş and Karakuş, "The logic of non-state armed groups survival in Syria: A contemporary framework of analysis," Studies in Conflict and Terrorism (2022).*

Thank you for bringing this to our attention, we had indeed not engaged sufficiently with literature on the dynamics of conflict within Syria in particular, and have incorporated a discussion of this literature in the manuscript (lines 62-87).

*Second, there is excellent research and reporting on Syria that the authors unfortunately do not draw upon. These sources could be leveraged to summarize the existing understanding of the fighting between Kurdish militias and ISIS, and, based on this, to highlight the significance of the manuscripts findings, either as complementary or contradictory to those existing understandings. Examples here include the followings books: Phillips, Frontline Syria: From Revolution to Proxy War (2020) (not cited); Oktav et al., Violent non-state actors and the Syrian civil war: The ISIS and YPG cases (2018) (not cited); Schmidinger, Rojava: Revolution, War and the Future of Syria's Kurds (2018) (cited, but no engagement with its contents); Schmidinger, Battle for the Mountain of the Kurds: Self-determination and ethnic cleansing in Rojava (2019) (not cited); Paasche et al. 2020, America's war in Syria: Fighting with Kurdish anti-ISIS forces (2020) (not cited); and Knights and van Wilgenburg, Accidental allies: The US–Syrian Democratic Forces partnership against the Islamic State (2021) (not cited). All of the above is in addition to substantial reporting from wire services like Reuters and newspapers like the Wall Street Journal, the Washington Post, the Guardian, the New York Times, and the like.*

While we do agree that the literature on civil war dynamics (mentioned in the previous point) was relevant, and for that reason included it in the revised version, we do not believe the literature mentioned here is directly pertinent to our paper. A majority of the sources mentioned relate to the role of the US in Syria – as we are not looking at the influence of external actors directly, it does not seem relevant to discuss this literature in depth. We do discuss in the paper the presence of external actors, as they have had an important impact on the conflict, however this is not the focus of the present paper. In particular, the sources that focus on the role of the US in Syria are:

- Phillips, Frontline Syria: From Revolution to Proxy War (2020)
- Paasche et al. 2020, America's war in Syria: Fighting with Kurdish anti-ISIS forces (2020)
- Knights and van Wilgenburg, Accidental allies: The US–Syrian Democratic Forces partnership against the Islamic State (2021)

Both books by Schmidinger provide a more historical account of either the wider Rojava region, or specifically Afrin. As mentioned by the reviewer, we do cite one of these sources, but again we do not believe an extensive discussion on the history of the region is pertinent in the paper.

Regarding the book by Oktav et al. (*Violent non-state actors and the Syrian civil war*), we appreciate the reviewer highlighting this. The discussion of non-violent armed groups, and ISIS and YPG in particular, is indeed highly relevant and highlights the importance of looking at the dynamics between these two groups. We have incorporated this discussion in lines 48-61.

*4. The manuscript does not provide information on how the analysis was conducted. On p.5, the authors write that they "map patterns of cooperation...and defection...from playing strategies against each other to observed actions recorded in the ACLED dataset." On p.7, they explain further that "The resulting patterns of defection were then mapped onto the event data for Rojava through a method of string-matching to identify recurring strategic interactions." I could not find any explanation, neither in the manuscript itself nor the supplementary material, of what method was used to do this. Thus, when the authors state on p.13 that "Territorial control proved to be the most significant variable in accounting for changes in behaviour, with temporal and geographical significance being largely due to the fact that both factors dictate shifts in territorial control," I am unable to assess on what basis the authors draw this conclusion. What methods are used to determine that "territorial control" is "the most significant variable"? Do the authors mean statistical significance? If so, further information is required, about the models used, the results of the estimation of these models, and so on. If no, further clarification about the substantive meaning of this sentence and other similar ones is needed.*

We have added some clarification to this in lines 334-336. However, we do explain how this was carried out and provide the full details and code in the Appendix. The approach used was first to play the strategies from the Prisoner’s Dilemma model against each other and record the resulting patterns of defection. Taking the ACLED data, we could then use a method of string-matching to identify these patterns within the conflict event data. As mentioned, we added some clarification, although from the first reviewer’s comments we understand that the methods were covered in sufficient detail.

*- The “systematic study of civil conflict” is not, contrary to the authors’ assertion “a remarkably recent field” (p.1).*

See answer to point 3 from Reviewer 1.

*- It is unclear what the authors mean by "The collective classification of civil conflicts has result in diverging theories" (p.1)*

We refer to the collective classification as opposed to disaggregation into types of conflict – for instance, one can categorise civil conflicts by identity and non-identity wars, as is done by Sambanis (2001 – Do Ethnic and Nonethnic Civil Wars Have the Same Causes?). The former includes ethnic conflict, which will have different causes to non-ethnic conflict. Therefore, looking at the causes of civil conflict in general we will be including diverging theories due to the different types of conflict that are included. We have clarified this in lines 17-18.

*- It is simply incorrect that "economists have highlighted abundance of natural resources" while "In contrast, political scientists have argued for the centrality of ethnic antagonism and existing disputes" (pp.1-2). In fact, scholars in both fields have advanced arguments that the authors of this manuscript mistakenly try to categorize as belonging within disciplinary boundaries.*

See answer to point 5 from Reviewer 1 – as cited, this distinction into three main strands of research comes from Kalyvas (2007 – Civil Wars).

*- It is incorrect that "Some argue that the strength of alliances varies between civil wars regardless of their nature" (citing Christia 2012; p.2). Christia's argument is that shifts in the balance of power across armed groups result in side-switching in civil wars.*

We wanted to highlight the point that it is not necessarily true that some types of conflict have stronger alliances because of the nature of the conflict – i.e. it could be assumed that ethnic conflicts for instance have stronger alliances as it is more based on identity. However, Christia (2012), mentions that “identity – be it racially, linguistically, religiously, or ideologically defined – appears to have no sustained causal role in the formation of alliances” (p.32), and later “notions of shared identity are not causes of alliance behavior but are employed instrumentally to justify the power-driven alliance decisions that are actually made by elites” (p.46). It is worth mentioning that she does go on to discuss the role of identity in the strength of subgroups.

*Materials and Methods: Important choices sometimes require additional explanation or reflection. For example, how does the restriction of the data to locations with 50 or more events (p.8) affect the findings? While understanding that the data might not allow for analysis of other locations, what kinds of biases might be expected in the results due to this limitation. Or, how does dropping events that exclusively involved violence against civilians (p.8) affect the results? Contrary to the authors' assertion that violence against civilians "do[es] not involve the two actors being considered here," entire areas of research in scholarship on civil wars study how violence against civilians is used strategically by armed groups in efforts to bring territory under their control (see Kalyvas, The Logic of Violence in Civil War (2006) and the subsequent research agenda that this book generated). What type of biases does excluding these events potentially introduce into the results?*

Regarding the biases introduced by the choice of data, we discuss the presence of different types of biases on a few occasions. The choice of locations to model is dictated by the availability of data – we were restricted to the locations that had sufficient data to model. We do acknowledge that biases will be present in terms of what events are recorded and what locations have a greater amount of reporting. This is already discussed in the manuscript, for instance lines 171-182 and lines 302-310.

Regarding violence against civilians, we agree with the reviewer that in not considering these events we may in fact be excluding instances of violence that were part of the conflict we are studying. However, we are not able to assertively place these events within the conflict, therefore we made the decision to only include events that decisively formed part of the Kurdish-ISIS conflict in Rojava. We have acknowledged this in more depth in lines 368-371.
