# Supplementary material for: Game-theoretic agent-based modelling of micro-level conflict: Evidence from the ISIS-Kurdish war
Source: PLoS One. 2024 Jun 5;19(6):e0297483. doi: 10.1371/journal.pone.0297483 (PMC11152260; doi:10.1371/journal.pone.0297483)
Supplement: Attachment — Submitted filename: Response to Reviewers.docx [file pone.0297483.s007.docx]

**Rebuttal Letter**

The authors would like to thank the reviewers for their fair reviews and detailed list of constructive suggestions. We greatly appreciate the opportunity to resubmit our revised paper for your renewed consideration. Below we respond to both the academic editor and reviewers’ comments and point to the corresponding changes we made in the paper. We mention the lines where these changes have been made, and have also submitted the new version of the manuscript with these changes highlighted.

## **Academic Editor**

*We encourage you to address the concerns and suggestions provided by the reviewers, particularly focusing on the:*

*i.* *clarification of purpose*

In order to clarify the purpose of our research, we have made significant changes to the abstract, introduction and conclusion sections. In the introduction in particular, we have focused on better framing our work within the context of existing literature, in this way highlighting our contribution, as well as being more explicit about our choice of method and case study. Below, we highlight a couple of short sections that we have added for the specific purpose of clarifying the purpose of the work:

From the introduction (lines 9-14):

“By integrating game theory into the agent-based framework, we scrutinise the evolving nature of violent events in Rojava, examining whether these incidents follow temporally and spatially stable patterns or vary significantly over time and location. This approach not only fills a notable gap in conflict literature but also provides a sophisticated understanding of the strategic behaviours characterising the Kurdish-ISIS conflict, offering insights into broader conflict dynamics and resolution strategies.”

From the abstract:

“Employing agent-based modelling aligned with Axelrod’s Prisoner’s Dilemma game-theoretic approach, the study provides a nuanced understanding of conflict dynamics in a highly volatile region, focusing on microdynamics of an intense dyadic strategic interaction between two near-equally-powered actors. The choice of Axelrod’s framework, though a classical approach, offers substantial insights due to its ability to model dyadic, equally-matched strategic interactions in conflict scenarios effectively.”

*ii.* *an explanation of your methodology, ensuring transparency and logical coherence in how the strategies from the Axelrod tournament and data from the ISIS-Kurdish conflict are applied and analyzed*

Our methodology is explained in the section titled Modelling Approach, and we provide further detail in the supplementary material, as well as a link to the repository containing the code for replication. We have added a section that reiterates the choice of modelling approach, highlighting why it is appropriate for this type of study and for the case of the conflict in Rojava in particular - this new section is from line 292 to 330.

As explained in lines 331-332, “we map patterns of *cooperation* (Dove) and *defection* (Hawk) from playing strategies against each other to observed actions recorded in the ACLED dataset” - by this we mean that we record the patterns that arise from playing Prisoner’s Dilemma strategies against each other, and match these recorded patterns to the conflict event data in the ACLED dataset.

*iii.* *elaborate on the choice of your case study, explaining its relevance and applicability for demonstrating your proposed analytical approach, and ensuring that its limitations and implications are transparently discussed.*

The case of Rojava presents a unique case study for a number of reasons: it presents a clear case of sustained dyadic interaction, with limited direct intervention from other actors. On top of this, the conflict is between ISIS and the Kurdish rebel forces - two non-state actors that are near-equally powered. Existing literature generally focuses on interactions that include at least one state actor, and as such often include some degree of asymmetry in terms of capacity. In choosing this conflict, we are able to look at the changing behaviour of two actors that are well matched in terms of power, and so more suited to a model such as the Prisoner’s Dilemma. We discuss the suitability of the chosen conflict both in the abstract and introduction.

We discuss limitations in a number of sections throughout the article. The limitations arise from a variety of sources; in particular, the availability of data, and that this data only records instances of conflict is discussed in lines 614-619:

“The reliance on datasets like ACLED, which primarily record instances of conflict, limits the ability to fully dissect these strategy combinations within the Rojava context. The need for more comprehensive data, particularly encompassing aspects of cooperation and negotiation, is evident to enhance understanding of de-escalation and conflict resolution strategies.”

We also discuss limitations in terms of bias in the data in lines 230-233:

“Given that the data covered relates to conflict, even a wide variety of sources cannot entirely remove the presence of data bias due to the political nature of events [65], the inherent difficulty in recording accurate data in conflict [35, 70, 71], and the fact that news reports often cater to a domestic audience and have a political agenda [72].”

## **Reviewer 1**

*1.* *The article needs a clearer purpose that “understanding the interaction between actors in conflict”. I.e., there needs to be something more than just a description of the data, for example predicting type of events/trajectories, and some comparison of a proposed approach against alternatives (e.g., predictions from a strategic model against autoregressive trends, for example).*

We would like to thank the reviewer for their comment, as this made us revisit how we were presenting our research and placing it in the context of existing literature. We have made changes particularly to the Abstract, Introduction and Conclusion to improve clarity throughout, as mentioned in our response to the academic editor’s first point. We clarified the fact that our contribution goes beyond the mere description of the data. In fact, the game-theoretic approach allows us to get a better understanding of the dynamics of interaction within this conflict. We agree that these points were not sufficiently clear in the previous version of the manuscript. This is highlighted in the conclusion, and we have made this clearer in lines 603-608:

“Our model reveals that the proportion of attacks carried out by each side is a more indicative measure of significant changes in the conflict than previously recognized patterns of strategic interaction. This finding challenges conventional approaches in conflict analysis, which often emphasise direct, bilateral engagements and immediate retaliations. Surprisingly, the model uncovers that a large majority of the behaviour consists of periods of one-sided violence, where retaliation, if present, is not immediate.”

We do not compare our model to approaches based on regression and more statistical analyses - the reason for this is the fact that, as mentioned in the article, the majority of these approaches focus on studying the causes and onset of conflict. Instead, we are interested in the dynamics during an ongoing conflict.

*2.* *The specific postulated contribution from game theory needs to be clarified. There are many analyses that seek to examine evidence for reciprocity in conflict levels (e.g., Goldstein and Pevehouse, Goldstein and Freeman), and could be seen as models to understand the effects of behaviors and possible interventions. The approach here seems to do something else – take a list of possible strategies in the Axelrod tournament, and then see if we can find evidence that the observed case resembles this. If if understand this correctly, the authors conclude that this cannot be done, since different strategies would lead to the same observed outcomes (i.e., pp.6-7). But the authors have not really clarified what it is they want to do and how this would be helpful.*

As mentioned in our response to the academic editor’s third point, part of our contribution comes from the types of actors involved in the chosen case study. The literature mentioned by the reviewer (Goldstein and Pevehouse, Goldstein and Freeman, etc.) looks at international conflicts and aggregates actors on a country level. Instead, we are looking at the interaction between two non-state actors that are equally matched, which lends itself to the application of the Prisoner’s Dilemma.

To address the point raised by the reviewer with respect to the objective of the study, we have significantly revised the abstract, introduction and conclusion. For instance, in lines 9-14 we emphasise the aim of the research:

“By integrating game theory into the agent-based framework, we scrutinise the evolving nature of violent events in Rojava, examining whether these incidents follow temporally and spatially stable patterns or vary significantly over time and location. This approach not only fills a notable gap in conflict literature but also provides a sophisticated understanding of the strategic behaviours characterising the Kurdish-ISIS conflict, offering insights into broader conflict dynamics and resolution strategies.”

*3.* *I appreciate that iterated PDs could be seen as a model of continuing conflict, which could escalate up and down, in dyadic interactions under receiprocity with different event sequences. But the application here is rather confusing since the authors talk about events as C and D, but then say that ACLED only allows them to measure Ds, and the data are split up by location and seemed to be binnned in time. The analysis simply seems to count number of attacks, and assume that responses must be place specific and have no memory of past interactions. I am not sure exactly what is done here or what we are learning from this. The results seem to be largely a description of the observed data, without any clear added value from a strategic perspective.*

The reviewer is correct in that the ACLED data only allows us to study instances of *defection*, which we discuss in the Modelling Approach section. However, we would like to point out that the analysis does not simply count the number of attacks - we look at the patterns present in the sequences of conflict events, and match these patterns to those that emerge from Prisoner’s Dilemma strategies. While we do carry out the analysis in specific locations, looking at the patterns within each location rather than across all recorded events, we also include a discussion of general trends in the data.

As mentioned above in our response to the reviewer’s first point, we do not provide only a description of the observed data. Our model reveals a lack of immediate retaliation in most instances, where a large majority of the behaviours observed are made up of one-sided violence. We also find that the proportion of attacks carried out by each side is a more indicative measure of significant changes in the conflict than previously recognized patterns of strategic interaction.

*4.* *I am also not sure why this is a particularly useful case to demonstrate a new approach to civil war interactions. It is a single dyadic interaction in a large civil war, unusual in that it is two non-state actors fighting rather than a recognized government (as is the case in typically very asymmetric civil wars). But presumably the interactions in such a setting will also be influenced by other actors in the conflict. If the aim is to examine a simple example, why not chose a conflict limited to two actors?*

We have addressed this question in our response to the academic editor’s third point. As the reviewer mentions, this case is unusual in that it involves two non-state actors. We also agree with the observations of the reviewer. We are focusing on actors that are closely matched in terms of power. This is indeed in contrast with an interaction between a state actor and a non-state actor, which is generally more asymmetric. We also chose to focus on the particular region of Rojava due to the sustained conflict between the two actors we study, and the lack of changes in alliances - as we have seen, shifting alliances are typical in the context of civil conflict, and the absence of this makes the chosen case more appropriate to study dyadic interaction. Therefore, to address the reviewer’s final question, by focusing on the region of Rojava, we have indeed chosen a conflict limited to two actors. We hope that the rewording of parts of the introduction and conclusions will contribute to clarify these points.

## **Reviewer 2**

We would like to thank Reviewer 2 for their time and for their positive feedback on the paper.
